# Oral lichen planus and HCV infection

**DOI:** 10.4322/acr.2020.210

**Published:** 2020-11-20

**Authors:** Jefferson R. Tenório, Alessandra Rodrigues de Camargo, Celso Lemos, Karem L. Ortega

**Affiliations:** 1 Universidade de São Paulo (USP), Faculdade de Odontologia, Patologia Oral e Maxilofacial, Departamento de Estomatologia, São Paulo, SP, Brasil; 2 Universidade Federal de Santa Catarina (UFSC), Departamento de Estomatologia, Florianópolis, SC, Brasil; 3 Universidade de São Paulo (USP), Faculdade de Odontologia, Departamento de Estomatologia, São Paulo, SP, Brasil

**Keywords:** Hepatitis C, Chronic, Lichen Planus, Oral

## Abstract

Chronic infection by hepatitis C virus (HCV) can lead not only to the development of hepatic cirrhosis, but also to the emergence of extra-hepatic manifestations (EHMs), such as oral lichen planus (OLP). Here, we describe a clinical presentation of massive, erosive OLP in an HCV-positive patient whose clinical management was difficult. Full remission was achieved after sustained virological response by using direct-acting anti-retrovirals. This case report demonstrates not only the importance of diagnosing EHMs for identification of HCV infection, but also the importance of controlling it for management of OLP and EHMs.

## INTRODUCTION

The infection by hepatitis C virus (HCV) has a characteristic immunological component in which there is a progressive loss of regulatory control over inflammation and increasing release of pro-inflammatory cytokines capable of leading to the destruction of hepatic parenchyma.^1^ ^,^ ^2^

Persistent immune activation and systemic inflammation have an impact on the progression of hepatic disease and development of extra-hepatic manifestations (EHMs) involving kidneys, eyes, musculoskeletal system, nervous system, skin and mucosas.^3^ Due to the scarcity of specific symptoms and signs of HCV infection, EHMs may be the first evidence of this infection.^4^

Lichen planus (LP) is one of the EHMs usually observed in individuals infected with HCV. In its idiopathic form, LP is described as a mucocutaneous condition affecting middle-aged adults, with a slight gender predilection for females.^4^ Overall, LP is a reaction immunologically mediated by antigens and especially orchestrated by T CD8+ lymphocytes, which promotes destruction of the keratinocytes in the basal layer of the epithelium.^5^ ^,^ ^6^

The first report of an association between LP and HCV was published in 1991.^7^ Since then, several hypotheses have been proposed to elucidate the mechanisms involved in the interaction between both conditions. However, their pathogenesis is not fully understood.^8^

The objective of the present case report is to describe the diagnostic process and treatment approach for an aggressive oral lichen planus (OLP) in a patient with HCV chronic infection.

## CASE REPORT

A 51-year-old male patient sought a specialized care center complaining of ulcerations in the mouth. Intense pain was making feeding, swallowing, speaking and oral hygiene difficult. He reported that oral lesions appeared 10 months earlier and were initially treated with acyclovir for 15 days, with no relief neither lesions’ improvement. His blood cell count showed a remarkable thrombocytopenia. A HCV serology using ELISA (MEIA, kit AxSYM® HCV version 3.0, Abbott Laboratories, North Chicago, Illinois, USA) was positive result, and was confirmed with qualitative PCR (Cobas Amplicor HCV Monitor TM test, version 2.0, Roche Diagnostic Systems, NJ, USA) (Genotype 1a). The patient was treated with ribavirin and pegylated alpha-interferon, according to the current clinical protocol and therapeutic guidelines of the Ministry of Health.

**Figure 1:**
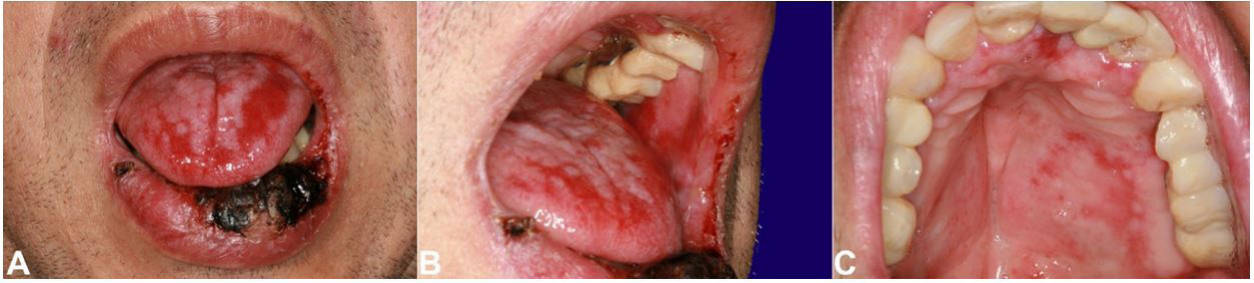
**A** – Clinical aspect of the oral lesions: Massive edema in the lower lip with extensive crust formation; whitish erythematous areas on the dorsum of the tongue; **B** – Whitish areas associated to erosive/ulcerative lesion were observed in the buccal mucosa bilaterally; **C** – Erosive areas in the hard palate.

After seven months of treatment, the patient sought our service because the oral lesions were progressively worsening. The physical examination showed that the lower lip was swollen and had extensive ulceration with crust formation (Figure 1A). These ulcerative erosive lesions were scattered all over the oral mucosa, being surrounded and interspersed by whitish lichenoid lines (Figure 11C), and purple scaling papules on the palms and dystrophic fingernails were observed (Figure 22B).

**Figure 2:**
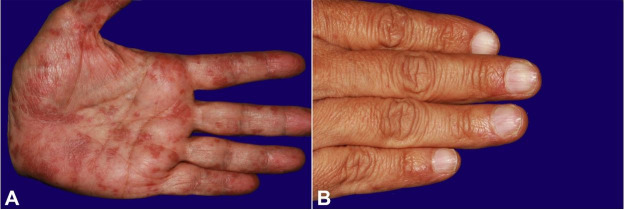
**A** – Aspect of the skin lesions. Polygonal purple papules in the palm of the hand; **B** – Dystrophic aspect of the fingernails.

**Figure 3:**
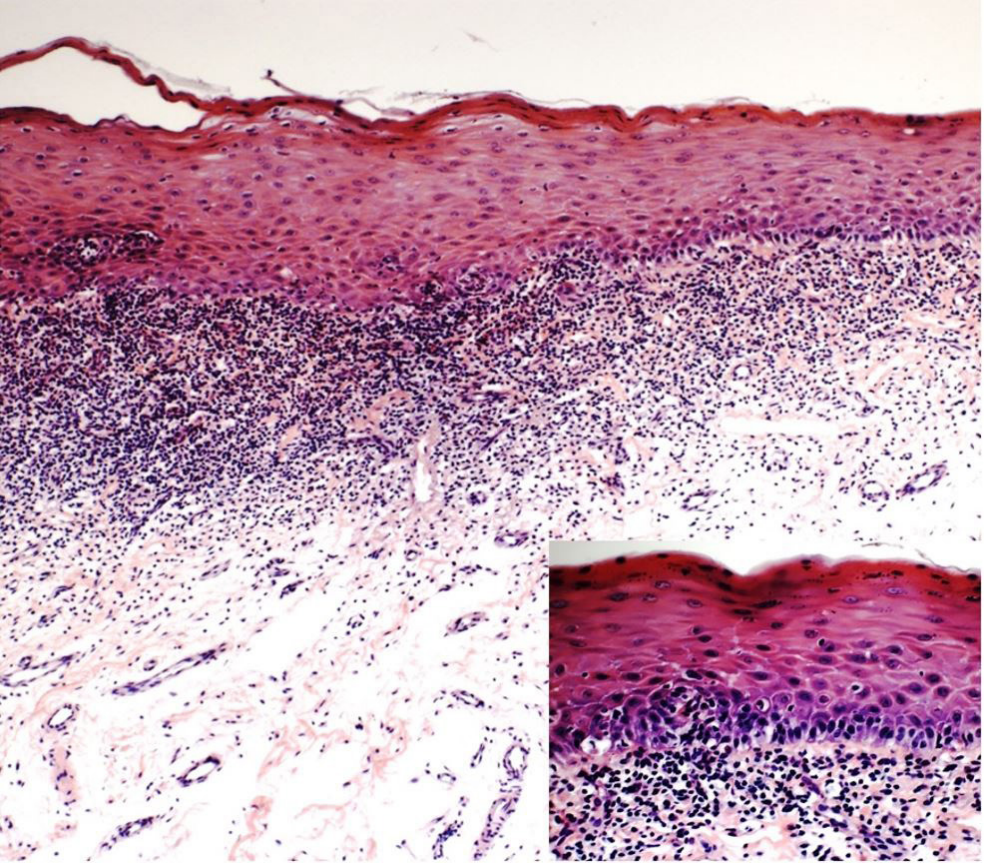
Photomicrograph of the buccal mucosa showing the band-like inflammatory infiltrate Note the intense and predominantly lymphocytic inflammatory infiltrate and presence of Civatte (apoptotic) body (HE, 400x magnification) (HE, 100x).

An incisional biopsy of the buccal mucosa was performed and histopathological examination showed a fragment of mucosa covered by hyper-parakeratinized stratified pavimentous epithelium, partially ulcerated, with intense and predominantly lymphocytic inflammatory infiltrate arranged in a juxta-epithelial band, which was compatible with the diagnosis of lichen planus (Figure 3).

**Figure 4:**
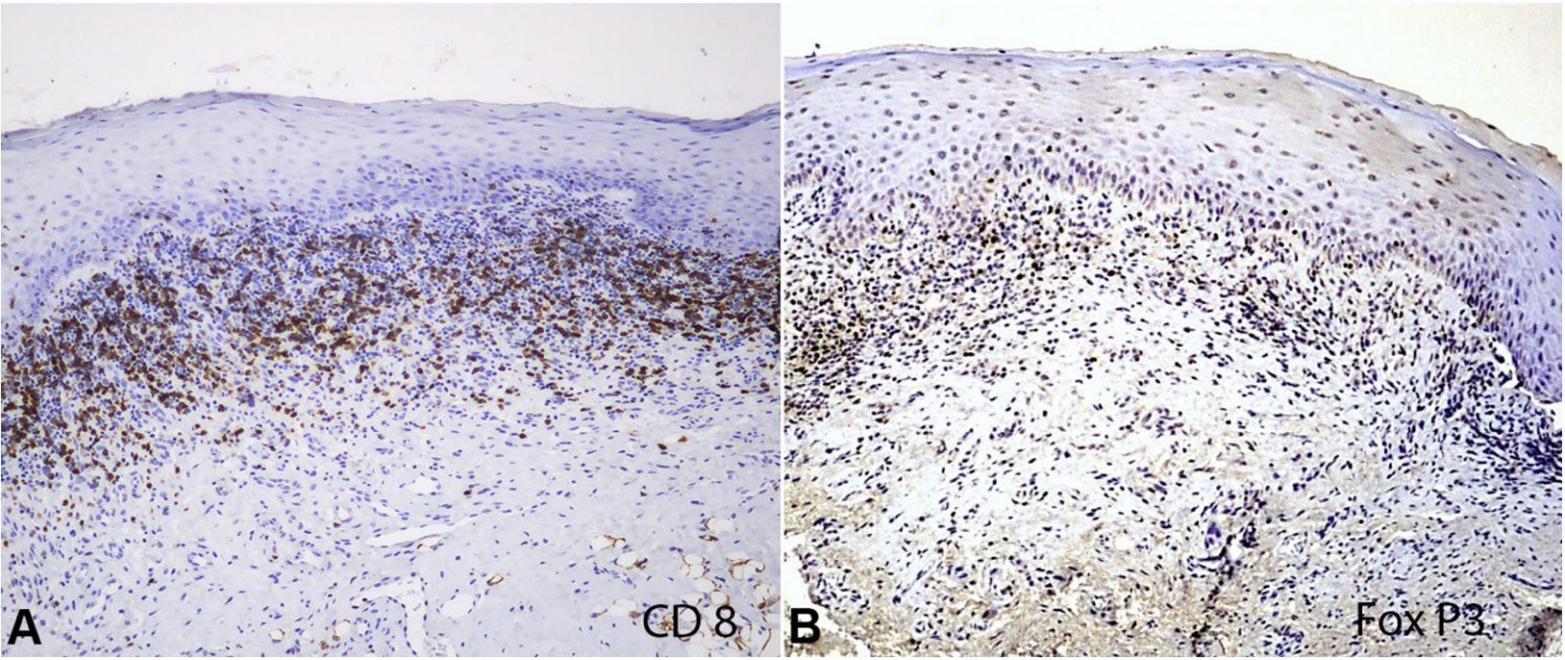
Photomicrograph of immunohistochemical reactions with primary antibodies anti-CD8 and anti-FoxP3 (40X and 100X respectively).

Additionally, immunohistochemical reactions with primary antibodies anti-CD8 (clone C8/144, DAKO, Glostrup, Denmark) and anti-FoxP3 (clone 236/E7, Abcam, Cambridge, UK) were also performed (Figure 4). Immune-positive cells were counted by using light microscope at magnification of 400x with LAS software V4.1. The counts of T CD8+ lymphocytes and T regulatory cells (Treg) were, respectively, 144 cells/mm^2^ and 50 cells/mm^2^.

Although the skin lesions were not biopsied, we believe these lesions were also manifestation of the lichen planus. Similar lesions are reported in the literature.^9^ ^,^ ^10^

Anti-nuclear antibodies (ANA), anti-DNA, anti-SSA and anti-SSB antibodies were analysed in order to rule out the possibility of systemic erythematous lupus, which were negative.

The initial treatment consisted of oral prednisone for 70 days (60 mg/day for 15 days, 40 mg/day for 10 days, 20 mg/day for 15 days and 5 mg/day for 30 days) and mouth rinsing with non-alcoholic solution of 0.05% clobetasol propionate (3x a day for 90 days). Significant improvement in the clinical picture was observed after 15 days of treatment with corticotherapy. The treatment of HCV was extended for 48 weeks, but the patient was considered a non-responder at the end of this period.

The oral lesions had alternating periods of remission and exacerbation during the 10-year follow-up, being clinically controlled by means of mouth rinsing with 0.05% clobetasol propionate and prednisone tablets.

The patient evolved to hepatic cirrhosis and entered the transplant list. Four years ago, the patient was treated with direct-acting anti-retrovirals (Sofosbuvir 400 mg/day, Daclatasvir 30 mg/day) for 12 weeks, which allowed sustained virological response (SVR) and significant improvement in the cirrhosis (compensated cirrhosis), resulting in his removal from the transplant list. The patient has been followed up since then (SVR) and until now he is in complete remission regarding the oral and cutaneous lesions. The patient is currently taking diuretics and non-selective beta-blockers for control of portal hypertension.

## DISCUSSION

Hepatic diseases are usually silent, with slow progression and being typically diagnosed when the organ’s function is broadly impaired. When HCV infection is the cause of hepatic dysfunction, there is a triggering of systemic inflammatory reactions with deregulation of the patient’s immune response, which leads to the emergence of EHMs. These EHMs can help the clinician diagnose HCV infection. In the present case report, the presence of oral lesions led us to perform a further investigation, which resulted in the diagnosis of chronic C hepatitis.

Another importance of EHMs is that the literature seems to point to inflammatory characteristics similar to those observed in the progression of hepatic disease caused by HCV and in the pathogenesis of autoimmune diseases, such as OLP. The enhanced CD8+ response against HCV, ^11^ facilitated by the decrease in the amount and efficacy of Treg cells (Foxp3+),^12^ ^,^ ^13^ is involved in both fibrosis extension and rapid progression to cirrhosis, such as in the pathogenesis of OLP.^14^ ^,^ ^15^ This is precisely the inflammatory picture found in the present case of oral lesions, as there was a ratio of CD8+ cells to FoxP3+ cells evidencing an imbalance in the mechanisms of immunological tolerance. The presence of a great amount of T CD8+ cells is also thought to account for the more severe clinical presentation of OLP, which seems to be more common in patients with hepatitis C.^16^ ^-^ ^19^

In the present case, the lesions observed in the patient were not only more severe, but also of difficult therapeutic control. Even using systemic and local corticosteroids, the episodes of recurrence had to be controlled for several years. As one believes that HCV is the factor leading to deregulation of the systemic immune response, the therapeutic inefficiency of interferon and ribavirin in countering viral replication may have been the factor promoting the OLP recurrence observed in the patient.

Despite the improvement in the patient’s clinical picture with the use of corticosteroids, the complete resolution of the OLP lesions only occurred after the SVR was achieved with the use of direct-acting antiretrovirals. There are several studies in the literature reporting that interferon (associated or not with ribavirin) can lead to both establishment and impairment of the clinical picture of OLP. ^20^ ^-^ ^22^ Some authors believe that this fact is due to the drug’s side effects.^20^ ^-^ ^22^ On the other hand, the inefficiency of the interferon-based treatment in countering the HCV viral replication and in leading to SVR might also be a factor promoting exacerbation of OLP. This second hypothesis was further supported after the publication of case reports associating the involution of OLP and other EHMs ^23^ ^,^ ^24^ to the SVR with the use of AAD, ^25^ ^-^ ^27^ including re-establishment of immune tolerance in the patients.^28^ ^-^ ^30^ These findings make us to suggest that the ideal treatment for cases of HCV-associated OLP should be coupled with the establishment of SVR.

This case report has demonstrated that it is important not only to diagnose EHMs for identification of HCV infection, but also to control it for management of OLP as EHMs.
